# Use of β‑blockers and risk of age‑related macular degeneration among hypertensive patients: An insight from The National Health and Nutrition Examination Survey

**DOI:** 10.3892/mi.2023.70

**Published:** 2023-01-30

**Authors:** Yili Luo, Jianpeng Liu, Wangqiang Feng, Da Lin, Guangwei Song, Mengji Chen, Haihua Zheng

**Affiliations:** 1 Department of Ophthalmology, The Second Affiliated Hospital of Wenzhou Medical University, Wenzhou, Zhejiang 325027, P.R. China; 2 Department of Pathology, The First Affiliated Hospital of Wenzhou Medical University, Wenzhou, Zhejiang 325000, P.R. China

**Keywords:** age-related macular degeneration, β-blocker, National Health and Nutrition Examination Survey, hypertension

## Abstract

Although age-related macular degeneration (AMD) is the leading cause of legal blindness, the treatment methods for AMD are limited. The aim of the present study was to examine the association between oral β-blockers (BBs) and the risk of developing AMD among hypertensive patients. For this purpose, a total of 3,311 hypertensive patients from the National Health and Nutrition Examination Survey were included in the study. The use of BBs and treatment duration data were collected using a self-reported questionnaire. AMD was diagnosed by gradable retinal images. Multivariate-adjusted survey-weighted univariate logistic regression was used to confirm the association between the use of BBs and the risk of developing AMD. The results revealed that the use of BBs exerted a beneficial effect (odds ratio (OR), 0.34; 95% confidence interval (95% CI, 0.13-0.92; P=0.04) in late-stage AMD in the multivariate adjusted model. When the BBs were classified into non-selective BBs and selective BBs, the protective effect in late-stage AMD was still observed in the non-selective BBs (OR, 0.20; 95% CI, 0.07-0.61; P<0.001). After accounting for treatment duration, long-term treatment with BBs (>6 years) was also found to reduce the risk of late-stage AMD (OR, 0.13; 95% CI, 0.03-0.63; P=0.01). In late-stage AMD, the long-term use of BBs was beneficial for geographic atrophy (OR, 0.07; 95% CI, 0.02-0.28; P<0.001). On the whole, the present study demonstrates that the use of non-selective BBs exerted a beneficial effect against the risk of late-stage AMD among hypertensive patients. Long-term treatment with BBs was also associated with lower risk of developing AMD. These findings may provide novel strategies for the management and treatment of AMD.

## Introduction

Age-related macular degeneration (AMD) is an eye disease whose incidence rate increases with age and leads to decreased central vision (1). AMD is the most common cause of legal blindness among the population aged >50 years in the western world (2). In the USA, the number of cases of legal blindness caused by AMD are greater than those caused by glaucoma, cataract and diabetic retinopathy in combination (3). AMD may have a severe impact on the quality of life of affected individuals. It is associated with an increased risk of functional disabilities, negative effects on daily activities, an increased risk of depression and a higher risk of developing cognitive impairments in older adults (4-6).

AMD has a variety of classification criteria. It is traditionally classified into the early and late stage based on color fundus images (7). Early-stage AMD is characterized by large drusen, retinal pseudo-drusen and pigmentary abnormalities. By contrast, late-stage AMD is divided into neovascular AMD (nAMD) and geographic atrophy (GA). Although AMD is the leading cause of legal blindness, the treatment methods available for late-stage AMD, particularly nAMD, are limited. The primary treatment for nAMD is based on the inhibition of vascular endothelial growth factor (VEGF) (8). Although several complement inhibitors are undergoing therapeutic clinical trials (ClinicalTrials.gov Identifiers: NCT05230537, NCT03364153 and NCT04465955), there are currently no effective therapeutic methods available for GA (9). In addition, there is also a lack of effective treatment strategies for preventing and delaying the progression of early- to late-stage AMD (7). Hence, the development of novel therapeutic agents for AMD is mandatory.

β-Adrenergic receptor (β-AR) blockers (BBs) are medications widely used in the treatment of heart diseases, such as hypertension, arrhythmias and heart failure. Previous preclinical studies have demonstrated a protective role of BBs against neovascularization. For example, propranolol treatment has been shown to reduce 50% of neovascularization in laser-induced choroidal neovascularization by reducing the release of VEGF (10). Reduced corneal neovascularization with downregulated levels of VEGF and cytokines was also observed following treatment with timolol in a murine corneal suture model (11). Hence, several clinical research studies have explored the possible association between BBs and AMD (12-19). However, some conflicting results have been reported. The studies by Klein *et al* (17) and Yeung *et al* (19) reported an increased risk of developing nAMD in patients treated with BBs compared to those not treated, while other studies, such as those by Traband *et al* (12), Kolomeyer *et al* (13), Thomas *et al* (15), Song *et al* (16) and Davis *et al* (18) found have no association between the use of BBs and nAMD development. Montero *et al* (14) suggested a beneficial effect of BBs against nAMD. Moreover, the majority of studies have focused on nAMD, and not on GA and early AMD. According to their selectivity for β-AR, the BBs used in clinical practice are divided into two main categories: Selective and non-selective BBs (20). The majority of research focuses on non-selective BBs. The association between the development of AMD and the use of selective BBs has not been reported to date, at least to the best of our knowledge. Thus, the present study investigated the association between different types of BBs and the risk of developing different stages of AMD using the data from the National Health and Nutrition Examination Survey (NHANES).

## Patients and methods

### Data source and ethics approval

All data used in the present study were obtained from the NHANES, which is a cross-sectional survey administered by the Centers for Disease Control and Prevention's National Centre for Health Statistics (NCHS) since 1999. It reflects the national status of health and nutrition in the USA. Since the present study employed de-identified information from the NHANES database approved by the institutional review board of the NCHS, the Ethics Committee of the Second Affiliated Hospital of Wenzhou Medical University (Wenzhou, China) granted the study an exemption from ethical review.

### Participants enrolled

**Figure 1:**
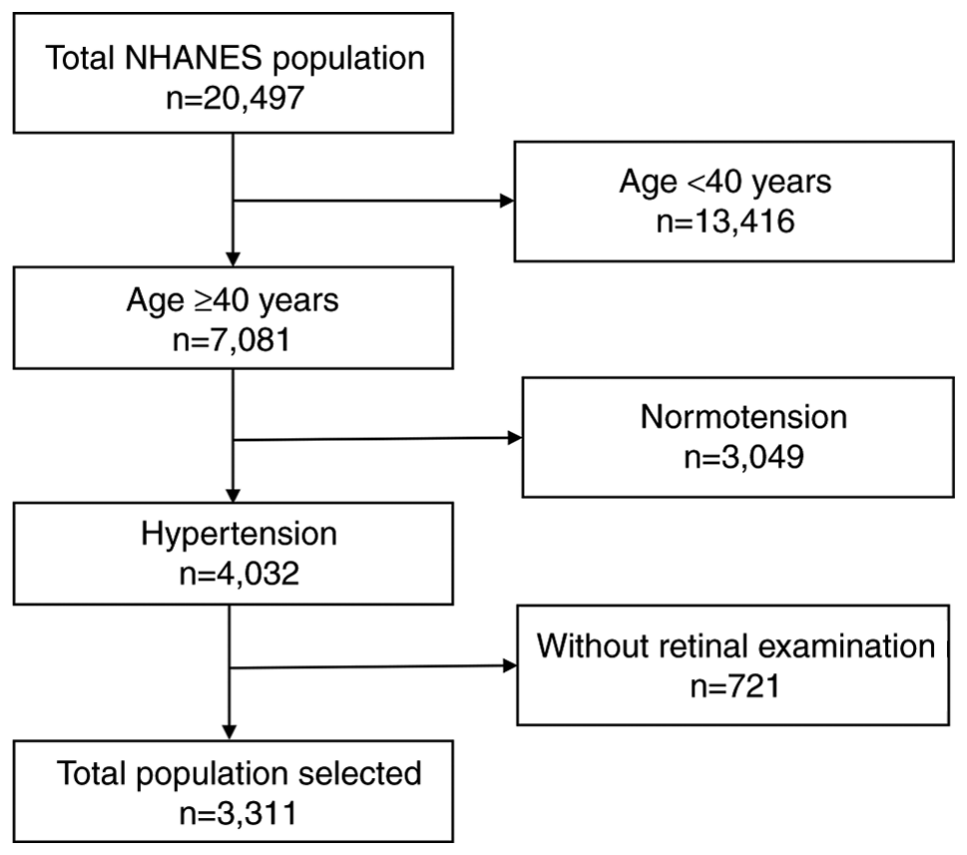
Flowchart demonstrating the number of patients excluded at each criterion and the ﬁnal inclusion cohort used for the study. NHANES, National Health and Nutrition Examination Survey.

As the retinal examination was only available in two NHANES cycles (2005-2006 and 2007-2008), participants were selected from these two cycles. Since BBs are mainly used in patients with hypertension, all the hypertensive participants selected were >40 years of age. Participants who responded ‘Yes’ to the question ‘have you ever been told by a doctor or other health professional that you had hypertension, also called high blood pressure?’ and those with an average systolic blood pressure ≥140 mmHg or average diastolic blood pressure ≥90 mmHg in the examination were defined as being hypertensive. In total, 20,497 participants were included from the two NHANES cycles; 13,416 participants were excluded as they were aged <40 years, among whom 4,032 had hypertension. A total of 3,023 participants were ﬁnally enrolled in the present study, for whom a complete retinal examination had been performed. A flowchart of the process used for the inclusion of participants is presented in Fig. 1.

### Classification of AMD

In the NHANES database, AMD was classiﬁed into three stages as follows: No AMD, early-stage AMD and late-stage AMD. The classification criteria were the following: Any large (≥125 µm) drusen, or retinal pseudodrusen or pigmentary abnormalities in the retinal examination were defined as early-stage AMD; any GA or exudative neovascularization in the retinal examination was defined as late-stage AMD. Those without any signs of early- or late-stage AMD in the retinal examination were considered as having no AMD. If both eyes were affected by AMD, data from the eye with the more severe stage of AMD were used.

### Use of BBs and treatment duration

The use of BBs was identiﬁed according to the self-reported prescription medications questionnaire (https://wwwn.cdc.gov/nchs/nhanes/continuousnhanes/questionnaires.aspx?BeginYear=2005). Non-selective BBs included propranolol, carvedilol, nadolol, sotalol, pindolol, labetalol, penbutolol and timolol. Selective BBs included nebivolol, metoprolol, atenolol, bisoprolol, acebutolol, and betaxolol. The duration of the use of BBs was also obtained from the questionnaire, which was divided into four quartiles as follows: First quartile, ≤2 years; second quartile, 2-4 years; third quartile, 4-6 years; fourth quartile, >6 years). The long-term use of BBs in participants was deﬁned as a BB treatment duration of >6 years.

### Other variables examined

Other variables included demographic characteristics, a history of comorbidities, health-related behaviors and the use of anti-hypertensive drugs. Data regarding age, sex, race, economic status and education level were collected under the category of demographic characteristics. A history of stroke, heart diseases, cancer or malignancy, diabetes mellitus, thyroid issues, glaucoma and diabetic retinopathy were collected with the history of comorbidities category. Health-related behaviors provided information about smoking and alcohol consumption. The use of anti-hypertensive drugs included the use of BBs and treatment duration, renin-angiotensin system inhibitors (RASIs), calcium channel blockers (CCBs) and diuretics. Participants with coronary heart disease, heart attack, angina pectoris or congestive heart failure were defined as having a history of heart disease. Participants with anemia or chronic bronchitis were defined as having a history of lung disease. Participants with glaucoma were identified by cup-to-disc ratios >0.6 in one eye. Participants with diabetes mellitus were identiﬁed using the following criteria: i) Fasting plasma glucose levels ≥126 mg/ml; ii) 2-h plasma glucose levels ≥200 mg/dl; iii) HbA1c ≥6.5%; and iv) answering ‘Yes’ to the question of ‘have you ever been told by a doctor or health professional that you have diabetes or sugar diabetes?’. Participants with diabetic retinopathy were identified by any signs of retinopathy (>14) on fundus images and by a diagnosis of diabetes mellitus. Dara regarding body mass index (BMI) and waist circumference had been measured during a physical examination at the time of the survey. Data on triglycerides (TGs), red blood cells (RBCs), white blood cells (WBCs), high-density lipoprotein (HDL) and platelet (PLT) levels of each participant had been obtained through a laboratory examination.

### Statistical analyses

Data were analyzed using a survey package in R software (version 4.1.3; http://r-survey.r-forge.r-project.org/survey/) with sampling weight following the complex sample design of NHANES. Continuous variables are presented as weight-adjusted mean ± standard error, and qualitative variables as weight-adjusted proportion ± standard error. ANOVA was used for the comparisons of means among multiple groups followed by Tukey's post hoc test. Survey-weighted univariate logistic regression was used to examine the association between different types of BBs and the various stages of AMD. A generalized additive model and natural cubic spline were used to explore the non-linear association between BB treatment duration and the risk of developing AMD. A multivariate model adjusted for age, sex, race, stroke history, heart disease history, thyroid disease history, glaucoma, RBCs and HDL was applied. The results are presented as odds ratios (ORs) with 95% conﬁdence intervals (95% CIs). The correlation between the use of BBs and the prevalence of AMD was calculated using Spearman's correlation analysis. The correlation between the use of non-selective BBs and the prevalence of AMD was also calculated using Spearman's correlation analysis. A value of P<0.05 was considered to indicate a statistically signiﬁcant difference.

## Results

### Characteristics of the participants enrolled

In total, 3,311 participants were enrolled in the present study. The participants with AMD tended to be older, of Caucasian or African-American origin, were married, had higher BMI and HDL levels, had low levels of RBCs, and had a history of heart disease, stroke and thyroid disease (Table I). In addition, a significant difference was found in the use of RASIs and BBs between participants with AMD and those with no AMD (Table I).

### Use of BBs and the risk of AMD in the hypertensive population

**Figure 2:**
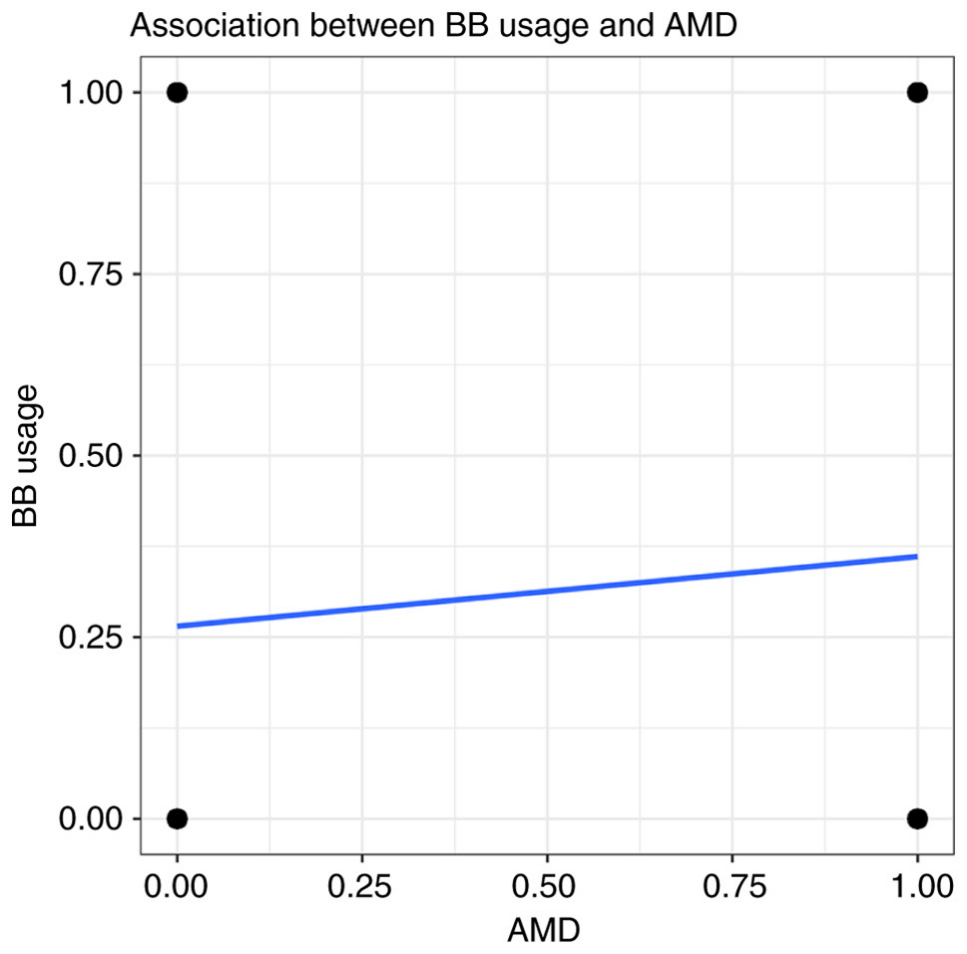
Spearman's correlation coefficient (Rho) and linear regression line between AMD and BB usage (Rho=0.06, P<0.05).

**Figure 3:**
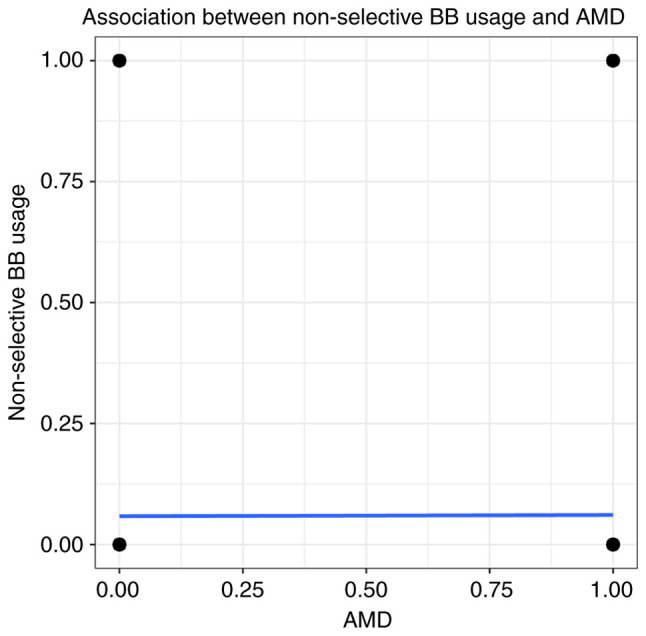
Spearman's correlation coefficient (Rho) and linear regression line between AMD and non-selective BB usage (Rho=0.07, P>0.05).

The association between the use of BBs and the risk of developing AMD was explored among all the participants. A significant correlation was found between the use of BBs and AMD (Rho=0.06, P<0.05) (Fig. 2). BB treatment increased the risk of developing AMD in the hypertensive population (OR, 1.49; 95% CI, 1.21-1.84; P<0.001) (Table II). When the BBs were categorized into non-selective and selective BBs, a significant association was found between the selective BBs and AMD (OR, 1.59; 95% CI, 1.29-1.97; P<0.001) (Table II). By contrast, no correlation was found between the use of non-selective BBs and AMD (Rho=0.07, P>0.05) (Fig. 3). However, no association was found between the use of BBs and AMD after adjusting for age, race, stroke history, heart disease history, thyroid disease history, glaucoma, RBCs and HDL (Table II).

### Use of BBs and the risk of early- and late-stage AMD in the hypertensive population

As the use of BBs did not have a significant effect on the risk of developing AMD following multivariate adjustment, the present study further explored whether the use of BBs was related to the different stages of AMD. Of note, there was no significant association between the use of BBs and the risk of early-stage AMD (Table III). Furthermore, no association was found after classifying the BBs into non-selective and selective BBs in the adjusted model. (Table III). However, the BBs exerted a beneficial effect (OR, 0.34; 95% CI, 0.13-0.92; P=0.04) against late-stage AMD in the multivariate adjusted model (Table III). The protective effect for late-stage AMD was observed in the non-selective BBs (OR, 0.20; 95% CI, 0.07-0.61; P<0.001) (Table III). By contrast, the selective BBs were not found to be significantly associated with late-stage AMD (OR, 0.46; 95% CI, 0.18-1.15; P=0.09) (Table III).

### BB treatment duration and risk of AMD

**Figure 4:**
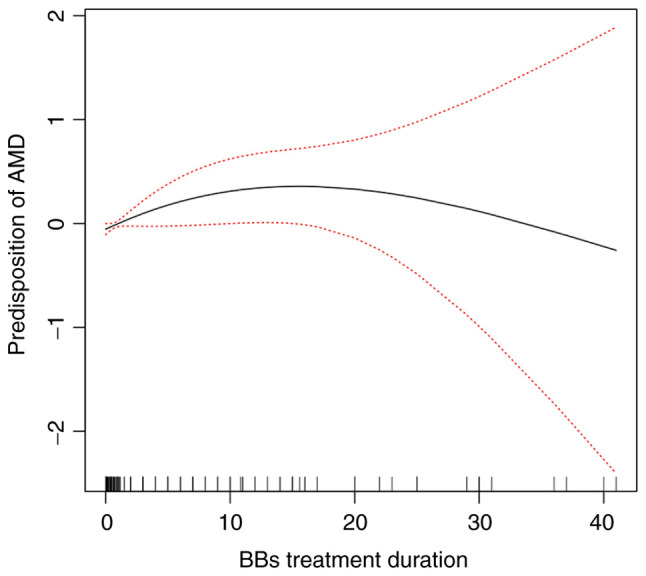
Dose-response association between the predisposition to AMD and BB treatment duration. The solid line is the smooth curve. The dashed lines are the 95% confidence intervals.

The aforementioned results indicated the protective effect of BBs against late-stage AMD. However, the potential cumulative effects of time were not previously considered in the literature, at least to the best of our knowledge. Hence, in the present study, the association between the use of BBs and the risk of developing AMD was further investigated. It was found that BB treatment duration had no association with the risk of developing AMD (OR, 0.98; 95% CI, 0.94-1.02; P=0.231; R^2^=0.114) (Table IV). In addition, no liner association was found between BB treatment duration and AMD using line regression analysis (OR, 0.998; 95% CI, 0.996-1.001; P=0.275; R^2^=0.061) (Table IV). A generalized additive model and natural cubic spline were introduced to examine the non-linear association. Thought the smoothing splines curve, the predisposition to AMD exhibited a trend to first increase, and to then decrease with the increasing treatment duration of BBs (Fig. 4). Thus, the BB treatment duration was we divided into four quartiles as follows: ≤2 years, 2-4 years, 4-6 years, and >6 years. Compared to the patients not on BB treatment, a decreased risk of developing AMD was only found in the last quartile of BB treatment duration (OR, 0.65; 95% CI, 0.43-0.98; P=0.04; R^2^=0.493) (Table V). The other groups did not exhibit a significant difference compared with the patients not on BB treatment (Table V). Similar results were found when examining the association between BB treatment duration and late-stage AMD. Only the fourth quartile of BB treatment duration exhibited a significant association with the risk of late-stage AMD compared with the BB non-users (OR, 0.13; 95% CI, 0.03-0.63; P=0.01) (Table V). Furthermore, with the increasing duration of BB treatment, a significant decrease in the magnitude of associations with the risk of late-stage AMD was observed (P for trend=0.048) (Table V). The other quartiles did not exhibit a significant association with the BB non-users (Table V). By contrast, for the early stage of AMD, no significant difference was found in BB treatment duration compared with the BB non-users (Table V).

### Long-term use of RASIs and different subtypes of AMD

The aforementioned results suggested that a BB treatment duration >6 years may decrease the risk of developing AMD. Therefore, the long-term use of BBs was defined as a BB treatment duration >6 years the present study. Since the previous assessments only focused on nAMD without considering GA and early-stage AMD, the long-term use of BBs was investigated in order to assess its influence on the different subtypes of AMD. Two major subtypes of early-stage AMD were mainly considered in the present study, including pigmentary abnormalities and soft drusen. However, there was no association between the long-term use of BBs and the two subtypes of early-stage AMD (Table VI). In late-stage AMD, the long-term use of BBs was a protective factor for GA (OR, 0.07; 95% CI, 0.02-0.28; P<0.001) (Table VI). However, it was considered that the result of the long-term use of BBs for GA was not reliable as the number of GA cases was very small. There was also no significant association between the long-term use of BBs and nAMD (Table VI).

## Discussion

In the present study, although there was insufficient evidence for the exact association between the use of BBs and AMD, a decreased association was found between the use of BBs and late-stage AMD among hypertensive participants from NHANES. The use of BBs, particularly long-term BB treatment, was found to exert a protective effect against late-stage AMD. Even though a significant protective effect of the long-term use of BBs against GA was found, due to the limited number of number cases of GA in the NHANES database, the outcome cannot be considered reliable. However, this result may provide the basis for the future clinical use of BBs and may guide future treatment strategies patients with AMD.

Several experimental studies have reported that β-AR plays a critical role in the development and progression of AMD, and suggest that BBs may be prophylactic drugs for nAMD. For example, propranolol was found to reduce retinal neovascularization and vascular leakage and was considered to downregulate retinal VEGF and insulin-like growth factor 1 expression (21). Carvedilol has also been demonstrated to modulate the expression of VEGF and hypoxia-inducible factor-1α induced by hypoxia (22). Dal Monte *et al* (23) also found that β-AR activation increased the expression of VEGF by increasing nitric oxide (NO) production, while β-AR blockers exerted the opposite effect by decreasing NO levels. BBs can reduce neovascularization. In addition, they can also improve the survival of retinal neurons. Betaxolol has been shown to exert neuroprotective effects in the retina by decreasing the expression of neuronal nitric oxide synthase (24). Betaxolol also reduces the death of neurons, reducing the calcium ion influx and sodium ion influx (25,26). In summary, BB treatment has been shown to exert therapeutic effects against neovascularization, which is the main pathophysiological mechanism of nAMD, and against the death of retinal neurons, which is the dominant mechanism of GA (27,28).

Although preclinical studies have indicated that BBs may be an effective treatment for AMD, clinical research on AMD and BBs has not yielded ideal results. A positive outcome was reported in the retrospective study by Montero *et al* (14). They found that the need for bevacizumab injections was decreased in patients with nAMD treated with oral systemic BBs compared to the BB non-users (14). However, that study was limited by small sample size. As hypertension is a risk factor for AMD, using participants not treated with BBs as the control group may possibly introduce confounding bias. A retrospective study involving the database of large national USA insurers found a opposite outcome (12). A comparator medication class with similar diseases was selected to address the bias. The effects of injections of anti-VEGF agents in hypertensive patients with BBs did not differ from those on hypertensive patients with CCBs (12). The aforementioned studies focused on the injection incidence. In comparison, other clinical studies have paid attention to the association between the risk of developing AMD and the use of BBs, and found negative results. For example, Davis *et al* (18) found no difference in the use of BBs between patients with GA and wet AMD. Thomas *et al* (15) also found there was no significant association between the use of BB and choroidal neovascularization in nAMD. However, the Beaver Dam Eye Study (BDES) revealed opposite results (17). BB treatment was associated with an increased 5-year incidence of exudative AMD over a 20-year period. That study also had limitations, such as not considering BB treatment duration. Furthermore, two longitudinal studies on BB treatment duration and AMD were conducted to explore the association between BB treatment duration and nAMD. Yeung *et al* (19) found that the continuous use of BBs was associated with a higher risk of nAMD compared with non-users. By contrast, Kolomeyer *et al* (13) reported that patients using BBs were signiﬁcantly less likely to develop nAMD at 90 and 180 days than patients using CCBs. The aforementioned studies concentrated on nAMD, while the study by Song *et al* (16) focused on GA; they found no significant association between BBs and nAMD.

Several researchers have examined the association between BBs and AMD. Although several of the outcomes were negative, further studies are required to fully elucidate the association. In the present study, the use of BBs was not found to be significantly associated with early-stage AMD and nAMD compared with non-users, reflecting the conclusions of some studies (12,13,15,18). However, long-term treatment with non-selective BBs had a protective effect against late-stage AMD. Since β-AR in the retina has an age-related overexpression and a super-sensitivity effect, it is possible that continuous BB treatment exerts a protective effect against AMD (29). However, the positive effect identified in GA in the present study was different from the study of Song *et al* (16), which found the use of BBs had no association with the incidence of GA. There were some explanations accounting for this difference. On the one hand, the participants enrolled were different. In the present study, hypertensive patients not treated with BBs were set as the controls, while other studies did not consider hypertension (14,15,16,18). On the other hand, the BB treatment duration may differ, as it was not considered in other studies (12-18). Thus, a prolonged BB therapeutic duration may result in a different outcome.

The present study has certain strengths. Firstly, hypertensive participants were enrolled to avoid confounding bias. In addition, a number of confounding factors aside from hypertension were adjusted for in the multivariate analysis. Secondly, the effect of different categories of BBs was investigated, while the majority of previous studies (12-16). only focused on non-selective BBs. Non-selective BBs block β1-AR and β2-AR, while selective BBs mainly inhibit β1-AR. However, all the subtypes of β-AR are expressed in retinal cells (30). The further examination of selective BBs in AMD is thus warranted. Herein, the association between the use of selective BBs and AMD was explored, revealing no significant association. Moreover, the association between the use of BBs and early-stage AMD was not explored in other studies (12-16,18,19). BDES reported no significant difference in the use of BBs and early-stage AMD. The results of the present study are in accordance with this outcome. Thirdly, the present study concentrated on the duration of BB treatment. Although short-term BB treatment (duration, <6 years) had no effect on AMD, there was a significant trend for decreasing the magnitude of associations of late-stage AMD with the increasing treatment duration of BBs.

However, there are limitations to the present study which should be mentioned. Firstly, all the data used were derived from NHANES, which is a cross-sectional study. The inherent flaws of cross-sectional design studies are unavoidable. Secondly, the use of BBs before or after AMD cannot be confirmed. Thirdly, the interactions among different anti-hypertensive drugs were not considered, which could lead to an overestimation of the protective effects of BBs.

In conclusion, the present study demonstrates that although the use of BBs did not affect early-stage AMD, the long-term use of BBs is a protective factor against the risk of AMD among hypertensive patients. However, the outcomes obtained need to be further validated in completely randomized or multi-center clinical trials involving the use of BBs and GA.

## Figures and Tables

**Table I:** Characteristics of the participants with AMD.

	AMD			
Characteristic	No AMD (n=2,998)	Early-stage AMD (n=272)	Late-stage AMD (n=41)	P-value
Demographics				
Age (years)	59.15±0.41	70.10±1.27	78.21±1.23	<0.001
Sex, female (%)	50.71±1.64	55.90±6.40	81.85±8.95	0.01
Race (%)				<0.001
Caucasian	76.34±2.61	88.83±3.32	97.94±2.14	
African-American	11.44±1.78	3.70±1.76	0.00±0.00	
Other	12.22±1.49	7.47±2.46	2.06±2.14	
Married or living with a partner (%)	69.75±1.54	57.28±6.88	30.40±7.79	<0.001
Education (high-school and above) (%)	80.36±1.08	72.13±7.15	66.58±12.28	0.981
Income at or above poverty	86.28±1.22	79.42±3.00	80.81±8.46	0.539
Health-related behaviors				
Smoking ≥100 cigarettes in whole lifetime (%)	16.61±2.08	15.87±4.28	10.11±10.19	0.395
Alcohol consumption in whole lifetime, ≥12 drinks (%)	53.76±1.61	63.41±3.03	37.46±6.74	0.266
Examinations				
BMI (kg/m^2^)	30.42±0.19	29.76±0.74	27.22±1.29	0.001
Waist circumference (cm)	104.14±0.47	104.68±1.68	99.03±3.35	0.658
Laboratory tests				
RBCs (10^6^ cells/µl)	4.73±0.02	4.63±0.05	4.34±0.04	<0.001
WBCs (10^3^ cells/µl)	6.96±0.09	7.11±0.25	7.12±0.27	0.669
PLT (10^3^ cells/µl)	268.45±2.14	258.60±8.87	245.96±11.32	0.079
HDL (mg/dl)	53.71±0.52	57.84±2.05	65.87±2.49	0.002
TG (mg/dl)	160.44±3.09	146.52±12.60	123.43±18.49	0.072
Disease history				
Heart diseases (%)	12.84±1.24	21.08±5.29	34.29±8.04	0.019
Stroke (%)	5.24±0.60	16.22±4.23	32.28±18.40	<0.001
Lung diseases (%)	16.56±1.44	12.56±3.11	16.92±7.62	0.111
Cancer or malignancies (%)	14.71±1.08	18.86±3.59	28.83±7.23	0.231
Thyroid issues (%)	14.78±1.34	23.64±4.03	36.27±11.58	<0.001
Diabetes mellitus (%)	17.58±1.18	15.90±3.06	26.89±7.79	0.106
Glaucoma (%)	3.48±0.62	5.76±3.27	9.71±6.96	0.191
Diabetic retinopathy (%)	13.75±1.09	17.88±4.49	0.00±0.00	0.944
Use of anti-hypertensive drugs				
RASIs (%)	46.00±1.12	47.86±5.85	59.80±9.05	<0.001
ACEI	28.84±1.10	27.77±3.86	52.58±10.12	0.213
ARB	18.12±1.33	20.10±4.35	17.07±6.09	0.431
BBs (%)	24.77±1.61	35.96±4.69	12.94±7.04	<0.001
Non-selective BBs	4.42±0.74	5.82±2.67	0.00±0.00	0.424
Selective BBs	20.42±1.38	30.55±3.97	12.94±7.04	<0.001
CCB (%)	17.00±1.27	17.09±3.07	34.66±10.02	0.018
Diuretics (%)	31.02±1.82	40.80±6.52	64.67±8.19	0.043

AMD, age-related macular degeneration; BMI, body mass index; RBC, red blood cell; WBC, white blood cell; PLT, platelet; HDL, high-density lipoprotein; TG, triglyceride; RASI, renin-angiotensin system inhibitor; ACEI, angiotensin-converting enzyme inhibitor; ARB, angiotensin receptor blocker; BBs, β-blockers; CCB, calcium channel blocker.

**Table II:** Association between AMD and the use of BBs.

	No adjusted		Multivariate adjusted^a^	
Parameter	OR (95% CI)	P-value	OR (95% CI)	P-value
BB use	1.49 (1.21-1.84)	<0.001	0.94 (0.71-1.25)	0.66
BB category				
Non-selective BBs	0.86 (0.48-1.57)	0.62	0.69 (0.37-1.33)	0.26
Selective BBs	1.59 (1.29-1.97)	<0.001	1.01 (0.77-1.33)	0.92

^a^Data were multivariate adjusted for age, sex, race, stroke history, heart disease history, thyroid disease history, glaucoma, red blood cells and high-density lipoprotein. AMD, age-related macular degeneration; BBs, β-blockers; OR, odds ratio; 95% CI, 95% confidence interval.

**Table III:** Association between different stages of AMD and various category of BBs.

	Early-stage AMD^a^		Late-stage AMD^a^	
Parameter	OR (95% CI)	P-value	OR (95% CI)	P-value
BB use	1.13 (0.85-1.5)	0.39	0.34 (0.13-0.92)	0.04
BB category				
Non-selective BBs	0.86 (0.45-1.64)	0.638	0.20 (0.07-0.61)	<0.001
Selective BBs	1.17 (0.89-1.53)	0.244	0.46 (0.18-1.15)	0.09

^a^Data were multivariate adjusted for age, sex, race, stroke history, heart disease history, thyroid disease history, glaucoma, red blood cells and high-density lipoprotein. AMD, age-related macular degeneration; BBs, β-blockers; OR, odds ratio; 95% CI, 95% confidence interval.

**Table IV:** Association between AMD and BB treatment duration in the different models^a^.

Model	OR (95% CI)	P-value	R^2^
Logistic regression	0.98 (0.94-1.02)	0.231	0.114
Linear regression	0.998 (0.996-1.001)	0.275	0.061

^a^Data were multivariate adjusted for age, sex, race, stroke history, heart disease history, thyroid disease history, glaucoma, red blood cells and high-density lipoprotein. Non-users were used as a reference. AMD, age-related macular degeneration; BBs, β-blockers; OR, odds ratio; 95% CI, 95% confidence interval.

**Table V:** Association between AMD and BB treatment duration in the generalized additive model.

	AMD^a^		Early-stage AMD^a^		Late-stage AMD^a^	
BB duration	OR (95% CI)	P-value	OR (95% CI)	P-value	OR (95% CI)	P-value
	Reference (non-users)		Reference (non-users)		Reference (non-users)	
<2 years	0.93 (0.59-1.47)	0.76	1.06 (0.69-1.64)	0.77	0.47 (0.12-1.84)	0.26
2-4 years	1.73 (0.87, 3.40)	0.11	2.03 (0.97-4.24)	0.06	0.40 (0.04-3.90)	0.40
4-6 years	1.08 (0.57, 2.02)	0.81	1.27 (0.72-2.24)	0.39	0.38 (0.04-3.64)	0.38
>6 years	0.65 (0.43, 0.98)	0.04	0.82 (0.54-1.27)	0.36	0.13 (0.03-0.63)	0.01
P for trend	0.94 (0.87, 1.03)	0.21	1.00 (0.92-1.09)	0.96	0.46 (0.40-0.99)	0.048

^a^Data were multivariate adjusted for age, sex, race, stroke history, heart disease history, thyroid disease history, glaucoma, red blood cells and high-density lipoprotein. Non-users were used as a reference. The R^2^ value of this model was 0.493. AMD, age-related macular degeneration; BBs, β-blockers; OR, odds ratio; 95% CI, 95% confidence interval.

**Table VI:** Association between AMD subtypes and long-term BB treatment^a^.

	OR (95% CI)	P-value
Early-stage AMD manifestations		
Pigmentary abnormalities	1.05 (0.72-1.52)	0.79
Any soft drusen	0.81 (0.56-1.15)	0.22
Late-stage AMD subtypes		
Exudative AMD	0.35 (0.05-2.48)	0.27
Geographic atrophy	0.07 (0.02-0.28)	<0.001

^a^Data were multivariate adjusted for age, sex, race, stroke history, heart disease history, thyroid disease history, glaucoma, red blood cells and high-density lipoprotein. Patients taking BBs for <6 years were used as the reference group. AMD, age-related macular degeneration; BBs, β-blockers; OR, odds ratio; 95% CI, 95% confidence interval.

## Data Availability

The datasets used and/or analyzed during the current study are available from the corresponding author on reasonable request.
